# Predicting the need for intubation in the first 24 h after critical care admission using machine learning approaches

**DOI:** 10.1038/s41598-020-77893-3

**Published:** 2020-12-01

**Authors:** Benjamin Ming Kit Siu, Gloria Hyunjung Kwak, Lowell Ling, Pan Hui

**Affiliations:** 1 Department of Anaesthesia and Intensive Care, Prince of Wales Hospital, Hong Kong, China; 2 Department of Computer Science and Engineering, The Hong Kong University of Science and Technology, Hong Kong, China; 3 Department of Anaesthesia and Intensive Care, The Chinese University of Hong Kong, Hong Kong, China; 4 Department of Computer Science, The University of Helsinki, Helsinki, Finland

**Keywords:** Medical research, Respiration, Data mining, Machine learning, Predictive medicine, Respiratory tract diseases, Therapeutics

## Abstract

Early and accurate prediction of the need for intubation may provide more time for preparation and increase safety margins by avoiding high risk late intubation. This study evaluates whether machine learning can predict the need for intubation within 24 h using commonly available bedside and laboratory parameters taken at critical care admission. We extracted data from 2 large critical care databases (MIMIC-III and eICU-CRD). Missing variables were imputed using autoencoder. Machine learning classifiers using logistic regression and random forest were trained using 60% of the data and tested using the remaining 40% of the data. We compared the performance of logistic regression and random forest models to predict intubation in critically ill patients. After excluding patients with limitations of therapy and missing data, we included 17,616 critically ill patients in this retrospective cohort. Within 24 h of admission, 2,292 patients required intubation, whilst 15,324 patients were not intubated. Blood gas parameters (P_a_O_2_, P_a_CO_2_, HCO_3_^−^), Glasgow Coma Score, respiratory variables (respiratory rate, S_p_O_2_), temperature, age, and oxygen therapy were used to predict intubation. Random forest had AUC 0.86 (95% CI 0.85–0.87) and logistic regression had AUC 0.77 (95% CI 0.76–0.78) for intubation prediction performance. Random forest model had sensitivity of 0.88 (95% CI 0.86–0.90) and specificity of 0.66 (95% CI 0.63–0.69), with good calibration throughout the range of intubation risks. The results showed that machine learning could predict the need for intubation in critically ill patients using commonly collected bedside clinical parameters and laboratory results. It may be used in real-time to help clinicians predict the need for intubation within 24 h of intensive care unit admission.

## Introduction

Endotracheal intubation is commonly performed in the critical care setting for airway protection or mechanical ventilation. However, emergent intubation is associated with higher risks than elective intubation^1^. A clinician needs to balance the risks of emergency intubation against the risks of delaying intubation in a patient who requires it, which is also associated with mortality^2^.

Except absolute indications such as upper airway obstruction, the decision and timing of intubation are often tailored for individual patients. To facilitate decision making, scoring systems help predict the need for mechanical ventilation during failed non-invasive ventilation or high flow oxygen therapy in patients with acute respiratory failure^3,4^. However, these scores have not been validated for patients without respiratory failure who require intubation for airway protection. Furthermore, their calculation relies on an accurate assessment of the P_a_O_2_/F_i_O_2_ ratio, which is not easily attainable in patients on variable performance oxygen devices.

Decision support systems aid clinical decisions by alerting clinicians and proposing treatments based on objective clinical data^5^. Databases such as Medical Information Mart for Intensive Care (MIMIC) have been used to build models that detect patients ready for discharge, predict the development of acute kidney injury^6,7^. Predictive model may provide an early warning to clinicians before conventional clinical signs manifest. Previous intubation prediction models have shown good performance up to 3 h prior to intubation, but longer lead time may facilitate time-sensitive intervention to prevent deterioration^8^. The objective of this study was to develop a tool utilizing bedside clinical and laboratory parameters at an intensive care unit (ICU) admission to predict the need for intubation within the next 24 h.

## Methods

### Data source

We performed a secondary analysis and built our predictive model on patients included in two databases, the Medical Information Mart for Intensive Care III (MIMIC-III) and the eICU Collaborative Research Database (eICU-CRD)^9,10^. The MIMIC-III database comprises data from 61,532 ICU stays at the Beth Israel Deaconess Medical Center between 2001 and 2012. The eICU-CRD is populated with > 200,000 admission data from a combination of many critical care units throughout continental United States from 2014 to 2015. These databases contain deidentified data, including high-resolution data of admission and discharge, diagnosis, data from monitors and laboratory results. The databases are released under the Health Insurance Portability and Accountability Act (HIPAA) safe harbor provision.

### Study population

We included all patients aged 18 and above and less than 90 in the eICU-CRD and MIMIC-III database who were not intubated before ICU admission. For patients with multiple ICU and hospital admissions, we only included data from the first ICU admission and first hospital stay. Exclusion criteria included patients with missing airway data or had do-not-resuscitate or do-not-intubate order within 24 h of ICU admission.

### Data

We collected demographics data (sex, age, specialty), physiological parameters (heart rate, blood pressure, respiratory rate, S_p_O_2_, GCS), laboratory variables (glucose, lactate, pH, P_a_CO_2_, P_a_O_2_), sequential organ function assessment (SOFA) score, airway device, ventilator data, oxygen therapy, and vasopressor use. Oxygen therapy was supplementary oxygen using any method other than endotracheal devices. These variables were selected because our aim was to develop a model based on data, observations and interventions which were consistently available at the time of ICU admission. The data points closest to the time of ICU admission were used. Patients without a full set of core parameters of heart rate, systolic blood pressure, diastolic blood pressure, mean arterial pressure, respiratory rate, and temperature within 1 h of ICU admission were excluded. Patients who had > 2 missing data of S_p_O_2_, Glasgow Coma Score (GCS), shock index, pulse pressure, glucose, P_a_O_2_, P_a_CO_2_, or HCO_3_^-^ within 2 h of admission were also excluded.

Missing data is a major limitation in database studies because it reduces sample size and introduces bias by patient selection and imputation^11–15^. For example, patients who are critically ill may have more blood tests, but this does not mean that more blood tests itself cause a higher severity of illness. Prediction models also perform better when missing data is addressed^12^. In addition, assumptions become increasingly accurate with more covariates. In this case, multiple imputations can help to overcome these biases^14,15^. Previously, algorithmic variants based on computationally intensive techniques such as Singular Value Decomposition, K-nearest neighbors (KNN), and relatively less complex methods such as mean and median imputation were used. More recently, imputation with deep learning models such as the Autoencoder (AE) has improved the performance of predictive models^13^. Autoencoder is a type of neural network that learns an appropriate representation of input with minimized reconstruction errors^16^. In this study, we used AE to impute missing data for S_p_O_2_, GCS, shock index, pulse pressure, glucose, P_a_O_2_, P_a_CO_2_, or HCO_3_^−^. These missing values were imputed by using data on gender, age, physiological parameters and laboratory variables recorded within 2 h of ICU admission. AE was constructed with a modified mean square error between the reconstructed layer and the input data based only on present features^17–20^. To do this, first we removed data points that were present to make them “missing” completely at random. Then we trained the AE to impute the missing data based on minimizing the mean square error between the value of the imputed data against the actual value of the removed features. The imputation processes of the training set and the test set were performed separately to avoid information leak into each dataset with Keras 2.2.4 and Tensorflow 1.15.0 in Python^21,22^. After partitioning of data, AE was conducted for each training and test set which converged around 0.05 error rate, and the datasets were used for machine learning classifiers. For comparison, we experimented with other forms of imputation but found that AE outperformed KNN imputation in outcomes of machine learning classification in our dataset (Supplementary Table S4 online). We also performed modelling on a subset of patients who did not have any missing data to assess efficacy of imputation.

### Model

Time of intubation was defined as the first record for airway of any tracheal device (endotracheal tube, tracheostomy, naso-endotracheal tube) or mechanical ventilation data. Patients who had time of intubation within 24 h from ICU admission were classified as intubated and the remaining patients were classified as non-intubated. Since the aim was to provide decision support for clinicians to assess the risk of the need for intubation upon ICU admission, we limited our prediction time window to within the first 24 h of ICU stay. Our rationale was an extension of the prediction window beyond 24 h whilst using only data at a single time point (ICU admission) would likely weaken the utility of the model since increases in lead time decrease model performance^8^.

We used random forest (RF) for our prediction task as it allows for conventional clinical interpretations of feature importance, along with comparisons using logistic regression (LR) with L2 penalty. Only data recorded before intubation time was used for predictive models. After unity-based data normalization, the entire intubated cohort of 2,292 patients was split into a training set and test set with a 6:4 ratio. The same number of non-intubated patients were used for the test set and all remaining patients were used for the training set. Due to the class imbalance, both models were trained with adjusting weights inversely proportional to class frequencies in the data. The training epochs and parameters were chosen based on error rate convergence and the best performance with shuffled and randomly selected data. To confirm the stability of overall process in random data partitioning, missing data imputation and machine learning classifiers were repeated 12 times. Since our aim was to develop a model that alerts physicians to patients with increased risk of needing intubation at ICU admission, optimal model performance was defined as the highest sensitivity without compromising specificity and accuracy. Sensitivity analysis was performed to find the best RF model threshold that achieved this goal. We used Scikit-learn 0.20.3 library for data pre-processing and models^23^. The primary objective was to predict the need for intubation within 24 h of ICU admission.

To assess the feature importance in the RF model, we used the local model-specific feature importance from the RF and the local model-agnostic SHAP (SHapley Additive exPlanation) values^23,24^. These complementary approaches facilitate the interpretation of feature evaluation. Feature importance was calculated from how much each feature (variable) contributed to decreasing impurity over the trees and datasets^23,25^. In contrast, SHAP values attribute to each feature the change in the expected model prediction when conditioning on that feature.

### Statistics

Median with interquartile range (IQR) were used to describe continuous variables. The Kolmogorov–Smirnov test was used to test for normality. Mann–Whitney U test was used for non-parametric comparisons between continuous variables. We used the chi-square test to compare discrete variables. Sensitivity, specificity, positive predictive value, negative predictive value, positive likelihood ratio, negative likelihood ratio and Area under the curve (AUC) of the receiver operating curve (ROC) were used to assess the performance of LR and RF. Model performance was also assessed separately with or without specifying non-surgical and surgical patients. Statistical analysis was performed with SciPy 1.2.2 library in Python^26^.

## Results

### Baseline demographics

**Figure 1:**
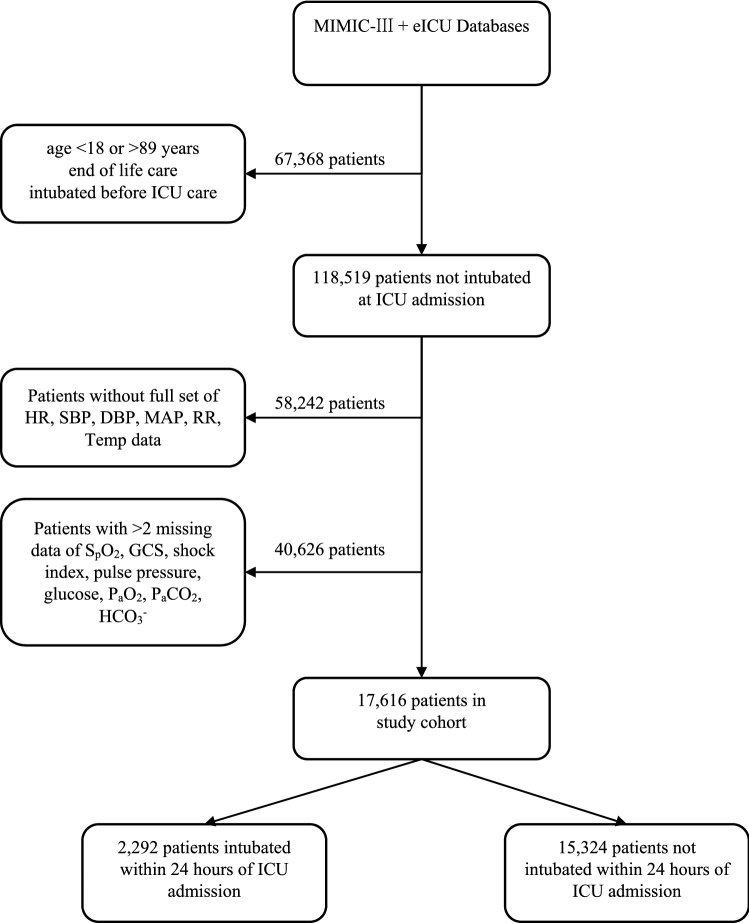
Flowchart for patient selection.

The combined database from eICU-CRD and MIMIC-III contained 185,887 patients. Of these, 17,616 patients fulfilled inclusion and exclusion criteria (Fig. 1). Up to 13% (2,292/17,616) of patients were intubated within 24 h of ICU admission. Of those who did not require intubation within 24 h, 5.1% (777/15,324) were intubated after 24 h. Baseline characteristics of our cohort are shown in Table 1. Baseline characteristics after imputation by AE are shown in Supplementary Table S1A and S1B online. The median S_p_O_2_ of patients given oxygen therapy was 97% (95 to 99).

**Table 1:** Baseline characteristics and outcomes of cohort.

	Intubated n = 2,292	Non-intubated n = 15,324	*p* value
Male (%)	1,299 (56.7)	8,301 (54.1)	0.0261
Age (years)	63 (52–74)	62 (50–74)	0.3576
SOFA score	6 (4–9)	4 (2–6)	< 0.001
**Specialty**			< 0.001
Medical (%)	1,204 (52.5)	9,374 (61.2)	
Surgery (%)	793 (34.6)	2,756 (18.0)	
Other/unspecified (%)	295 (12.9)	3,194 (20.8)	
SBP (mmHg)	121 (104–141)	125 (108–143)	< 0.001
DBP (mmHg)	64 (53–78)	68 (57–80)	< 0.001
MAP (mmHg)	82 (69–95)	84 (72–97)	< 0.001
Heart rate (bpm)	93 (79–111)	89 (75–105)	< 0.001
Shock index	0.78 (0.62–0.96)	0.71 (0.57–0.89)	< 0.001
Respiratory rate (breaths/min)	21 (16–27)	19 (16–24)	< 0.001
S_p_O_2_ (%)	98 (94–100)	98 (95–99)	0.3940
Temperature (^o^C)	36 (36–37)	36 (36–37)	< 0.001
GCS	15 (13–15)	15 (14–15)	< 0.001
Random glucose (mg/dL)	137 (109–175)	134 (107–180)	< 0.001
P_a_O_2_ (mmHg)	103 (72–190)	88 (74–102)	< 0.001
P_a_CO_2_ (mmHg)	41 (34–50)	40 (33–48)	< 0.001
HCO_3_^-^ (mmol/L)	23 (19–26)	23 (20–26)	< 0.001
Oxygen therapy (%)	405 (17.7)	2,467 (16.1)	0.0616
Vasopressor (%)	111 (4.8)	419 (2.7)	< 0.001
Time to Intubation (hour)	4.53 (1.15–11.14)	–	–
ICU LOS (days)	4.06 (2.05–8.13)	1.65 (0.96–2.86)	< 0.001
ICU mortality (%)	292 (12.7)	274 (1.8)	< 0.001

All values are reported in median and interquartile range unless specified.

*SOFA* sequential organ failure assessment, *SBP* systolic blood pressure, *DBP* diastolic blood pressure, *MAP* mean arterial blood pressure, *GCS* glasgow coma score, *ICU* intensive care unit, *LOS* length of stay.

### Model performance

**Figure 2:**
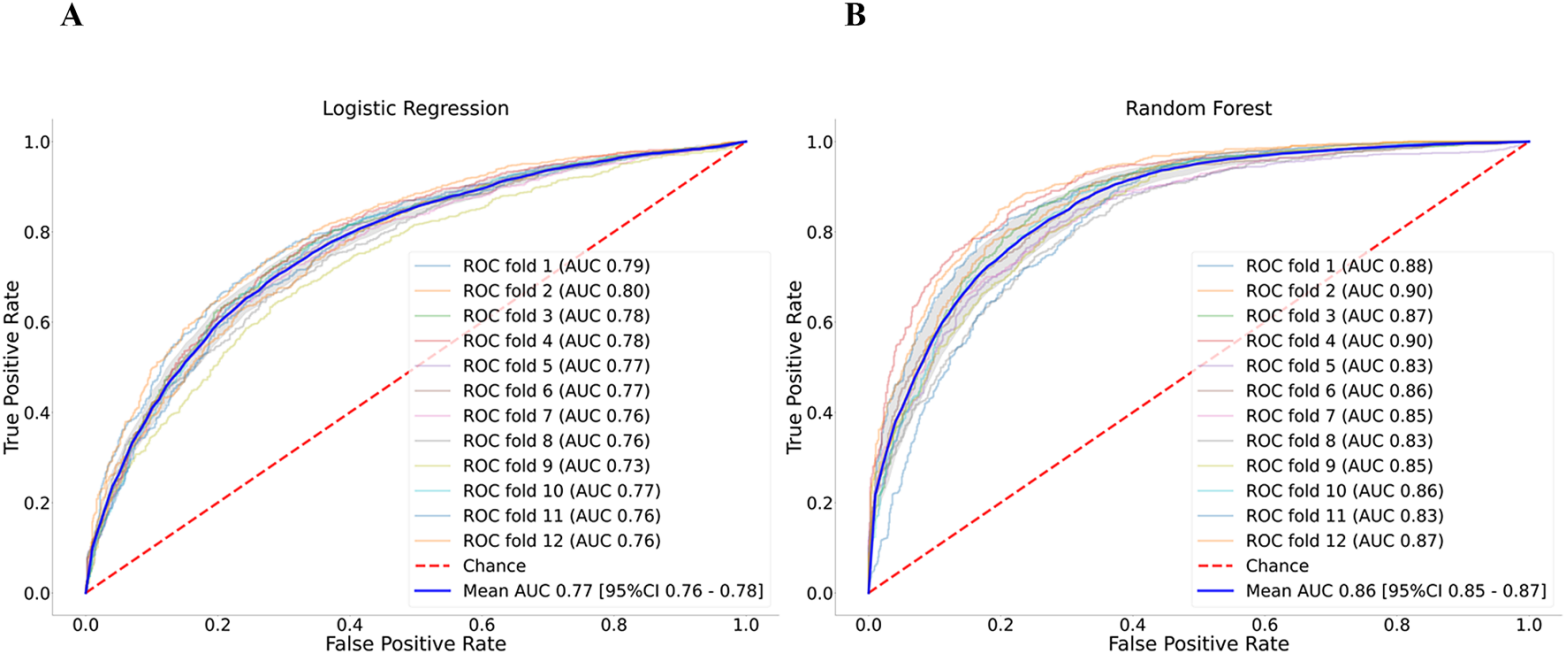
ROC curves of models to predict intubation.

**Figure 3:**
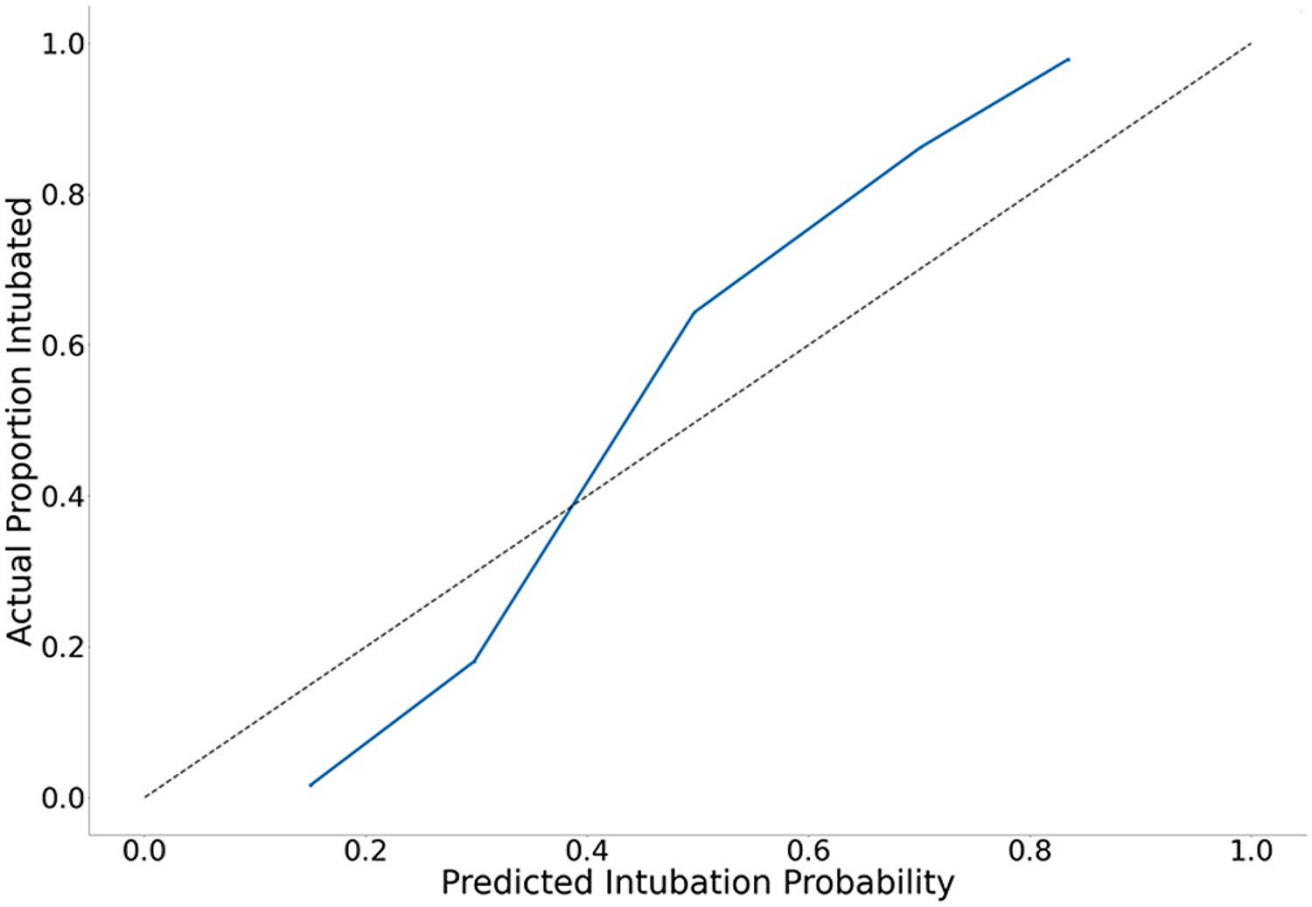
Calibration of random forest to predict intubation.

**Figure 4:**
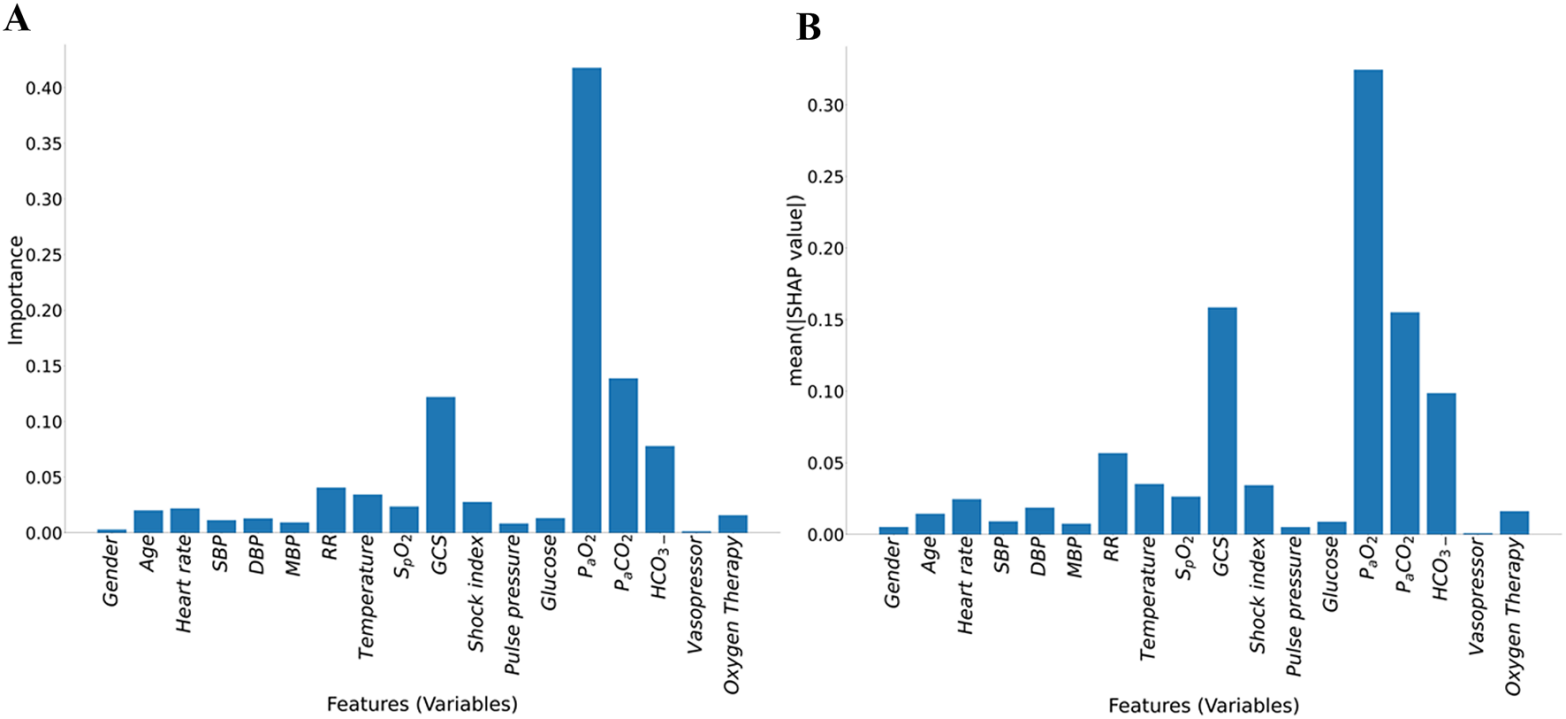
Feature importance and Shapley values of variables from random forest.

Our final model with RF had AUC 0.86 (95% CI 0.85–0.87) to predict intubation within 24 h of ICU admission. Comparatively, LR model only had AUC 0.77 (95% CI 0.76–0.78). Sensitivity analysis of our RF model using different thresholds is shown in (Supplementary Table S5 online). Overall, a threshold of 0.4 resulted in the best compromise in sensitivity of 0.88 (95% CI 0.86–0.90), specificity of 0.66 (95% CI 0.63–0.69), accuracy of 0.77 (95% CI 0.76–0.78), and AUC of 0.86 (95% CI 0.85–0.87). Other performance indicators of the RF model is shown in Table 2. The ROC curves for each fold and mean are shown in Fig. 2. RF model showed good calibration over the whole range of intubation risk prediction (Fig. 3). Analysis of feature importance is shown (Fig. 4 and Supplementary Table S2 and S3 online). The SHAP values and feature importance from random forest showed similar and consistent patterns. Gender, pulse pressure and use of vasopressor were relatively less important features. The performance of LR and RF in a smaller cohort of 2,345 patients without missing data is consistently lower than models trained on imputed data (Supplementary Table S4 online).

**Table 2:** Random Forest model performance.

	AUC	Specificity	Sensitivity	NPV	PPV	NLR	PLR
Fold 1	0.88	0.74	0.85	0.84	0.77	0.20	3.32
Fold 2	0.90	0.74	0.89	0.87	0.78	0.15	3.48
Fold 3	0.87	0.68	0.89	0.87	0.74	0.16	2.83
Fold 4	0.90	0.72	0.89	0.87	0.76	0.15	3.18
Fold 5	0.83	0.59	0.89	0.84	0.68	0.19	2.17
Fold 6	0.86	0.71	0.83	0.81	0.74	0.24	2.90
Fold 7	0.85	0.69	0.82	0.80	0.73	0.26	2.68
Fold 8	0.83	0.62	0.87	0.82	0.69	0.21	2.25
Fold 9	0.85	0.57	0.92	0.88	0.68	0.14	2.15
Fold 10	0.86	0.57	0.94	0.90	0.69	0.11	2.19
Fold 11	0.83	0.67	0.84	0.81	0.72	0.24	2.52
Fold 12	0.87	0.68	0.89	0.86	0.73	0.16	2.76
Mean	0.86	0.66	0.88	0.85	0.73	0.18	2.72

*AUC* area under the curve, *PLR* positive likelihood ratio, *PPV* positive predictive value, *NLR* negative likelihood ratio, *NPV* negative predictive value.

## Discussion

Using data derived from 17,616 patients, we developed a model which could predict the need for intubation in critically ill patients within 24 h of ICU admission with sensitivity 0.88, specificity 0.66 and AUC 0.86. The model only uses bedside parameters that are routinely available at the time of critical care admission. Our predictive model may be used clinically to alert physicians on patients at increased risks of needing intubation within 24 h of ICU admission without additional workload for medical or nursing staff.

Risk factors associated with the need for intubation in specific populations such as patients with inhalation injury or acute poisoning have been reported^27,28^. However, intubation risk prediction models have generally focused on patients with respiratory failure^3,4,8,29^. Our model had better performance for both non-surgical and surgical critically ill patients when compared to single center studies on patients with respiratory failure admitted to surgical and trauma ICUs^29,30^. In another study also using MIMIC-III data, Ren et al*.*’s gradient boosting model had AUC 0.89 (95% CI 0.87 to 0.91) to predict intubation with a lead time of 3 h^8^. Although it had better performance than our model, it required at least twice as many predictive parameters. More than half were of these parameters were based on laboratory tests, which may not be readily available at admission. Detailed handling of this missing data and effects of imputation was not reported in their study. Furthermore, parameter values from two time points were used in their model. Instead of a 3 h prediction window, our model risk predicts intubation within the first day of ICU stay using commonly available physiological data and point of care test results at a single time point (ICU admission). Another advantage of our model is external validity based on model training of a 17,616 patient cohort combined from MIMIC-III and eICU-CRD databases consisting of different medical, surgical and mixed ICUs. Our model was also internally validated by random selections of patients into 12 different training and test cohorts. We showed that the model generated is stable across these training cohorts, which reduced the chance of noise or overfitting. It had consistent performance across the entire range of intubation risk prediction (Fig. 3).

Proposed scoring systems such as HACOR and ROX predict the need for intubation in patients with respiratory failure treated with non-invasive ventilation (NIV) and high flow nasal cannula (HFNC), with AUC 0.88 (95% CI 0.85–0.90) and AUC 0.74 (95% CI 0.64–0.84), respectively^3,4^. Although the performance of our RF model’s AUC 0.86 (95% CI 0.85–0.87) is within this range, there are important differences. First, HACOR and ROX models are only applicable to patients with severe respiratory failure who are already on NIV or HFNC. In contrast, patients with respiratory failure only accounted for a small proportion in our cohort, as reflected by the low prevalence of those who were given oxygen therapy (16.3%, 2,872/17,616). Nevertheless, we found that our model did not lose predictive performance even when expanding the cohort from non-surgical to include all ICU patients (Supplementary Table S6). There was consensus between the model using all patients and the model for only non-surgical patients, which balances local system performance and generalization^31^. Furthermore, our model had better sensitivity 0.88 (95% CI 0.86–0.90) but comparative specificity 0.66 (95% CI 0.63–0.69) to HACOR (sensitivity 0.62 and specificity 0.93) and ROX (sensitivity 0.70 and specificity 0.72)^3,4^. Second, HACOR and ROX utilize P_a_O_2_/F_i_O_2_ ratio in risk prediction as accurate F_i_O_2_ can be obtained in patients NIV and HFNC. In contrast, F_i_O_2_ component cannot be accurately estimated in patients on variable performance oxygen devices. Thus we built our model on the absence or presence of oxygen therapy rather than dependence on reliable P_a_O_2_/F_i_O_2_. Third, the ROX index was only shown to be useful 12 h after initiation of HFNC.

The most important features in our model included blood gas parameters, GCS and RR, which are similar to previous intubation risk prediction models^3,4,8^. We used two independent feature assessments, feature importance from random forest and SHAP showed consistent important feature patterns. Since GCS, RR and blood gas results are important clinical features of the neurological and respiratory assessment, it is not a surprise that they are the most contributing features of an intubation prediction model. Yet counterintuitively, we found that intubated patients had a higher median P_a_O_2_ prior to intubation compared to those who did not require intubation. We postulate this may be because patients who appeared more unwell were perhaps more likely to be given supplementary oxygen. Indeed, our finding of elevated P_a_O_2_ in patients who required intubation is consistent with Ren et al.’s intubation prediction model for patients with respiratory failure^8^. In contrast, the importance of S_p_O_2_ as a predictor of intubation risk was relatively low. This is different to Politano et al*.*’s model on surgical and trauma patients with respiratory failure which utilizes S_p_O_2_^29^. In a neonatal model, intubation for respiratory decompensation was also modelled by reduced S_p_O_2_^32^. Reduced importance of S_p_O_2_ in our model is likely because the proportion of patients with respiratory failure in our cohort is relatively small. Furthermore, since the goal of oxygen therapy is to maintain oxygenation, it’s possible that only a minority of patients with severe respiratory failure who were not intubated before ICU admission would manifest abnormal S_p_O_2_ on arrival to ICU. Indeed, patients who were given oxygen in our cohort had median S_p_O_2_ of 97%. Again, the surprisingly low importance of S_p_O_2_ is not unique to our model, as S_p_O_2_ was found to be of lower importance than urine output or age in Ren et al*.*’s model^8^.

Often machine learning models are studied using a single-centered specific time frame and potentially biased retrospective data, and have been proposed as tools that can be implemented in practice without careful consideration in the medical field^33^. In this project, we demonstrated the scalability, generalisability and clinical interpretability of this model using multicenter databases and easily collectable bedside parameters at ICU admission, and taking into account the effects of interpolation of missing values and comparisons of performance with multiple evaluation indicators.

This study has several key limitations. Firstly, data extracted from ICUs in the United States may not reflect international practice. Nevertheless our model was derived from a large multicenter derivation cohort of nonspecific critically ill patients. Secondly, the complexity of the RF makes an analysis of the construct counterintuitive to the clinician. But most of the important features were clinically relevant. Thirdly, we imputed missing data, which could affect the outcomes of our models. However, we showed that baseline characteristics remain largely unchanged after missing data imputation (Supplementary Table S1A and S1B online). Therefore, even if patients had missing data, imputation may be used to fill missing data and still provide risk prediction using our model. Fourth, we were unable to utilize diagnosis into the models as diagnostic code were performed later in the ICU stay. Nevertheless, future intubation risk models may be enriched by addition of provisional diagnosis recorded at ICU admission. Fifth, we limited our prediction time to within 24 h. It is possible that some patients who actually required intubation were only intubated after 24 h due to delay. However this bias effect is likely minimal in our cohort since only 5.1% of patients classified as non-intubated required intubation after the initial 24 h. Finally, certain clinical parameters, such as the paradoxical movement of abdominal muscles, are associated with respiratory failure^34^. Unfortunately it was not possible to consistently extract physical examination findings from the databases. Further studies may perform analysis of clinical progress notes to increase the performance of prediction models.

## Conclusion

We developed a tool to predict the need for intubation in critically ill patients within first 24 h of admission to ICU. Since it only uses simple routinely captured bedside parameters, it may be used in real-time to the predict need for intubation upon ICU admission.

## Supplementary information

Supplementary Information

## Data Availability

The datasets analysed during the current study are available in the PhysioNet repository, MIMIC-III: https://physionet.org/content/mimiciii/1.4/ and eICU-CRD: https://physionet.org/content/eicu-crd/2.0/. The datasets generated during the current study along with scripts to create the analyses and processed datasets are available in the Github repository, https://github.com/ucabhkw/INTML20.
