# Supplementary material for: Predicting the need for intubation in the first 24 h after critical care admission using machine learning approaches
Source: Sci Rep. 2020 Dec 1;10:20931. doi: 10.1038/s41598-020-77893-3 (PMC7708470; doi:10.1038/s41598-020-77893-3)
Supplement: Supplementary file 1 — Supplementary Information [file 41598_2020_77893_MOESM1_ESM.docx]

**Title:** Predicting the need for intubation in the first 24 hours after critical care admission using machine learning approaches

**Authors:** Benjamin Ming Kit Siu^1*^, Gloria Hyunjung Kwak^2*†^, Lowell Ling^3†^, Pan Hui^2,4^

^1^Department of Anaesthesia and Intensive Care, Prince of Wales Hospital, Hong Kong, China

^2^Department of Computer Science and Engineering, The Hong Kong University of Science and Technology, Hong Kong, China

^3^Department of Anaesthesia and Intensive Care, The Chinese University of Hong Kong, Hong Kong, China

^4^Department of Computer Science, The University of Helsinki, Helsinki, Finland

* Co-first authors. † Corresponding author

**Supplementary Tables and Figures**

**Supplementary Table S1A.** Baseline characteristics (median) of cohort before and after missing data imputation

|  | Before Imputation | | | After Imputation | | |
| --- | --- | --- | --- | --- | --- | --- |
|  | Intubated  n = 2,292 | Non-intubated  n = 15,324 | *p* value | Intubated  n = 2,292 | Non-intubated  n = 15,324 | *p* value |
| Male (%) | 1,299 (56.7) | 8,301 (54.1) | 0.0261 | 1,299 (56.7) | 8,301 (54.1) | 0.0261 |
| Age (years) | 63 (52 – 74) | 62 (50 – 74) | 0.3576 | 63 (52 – 74) | 62 (50 – 74) | 0.3576 |
| SBP (mmHg) | 121 (104 – 141) | 125 (108 – 143) | < 0.001 | 121 (104 – 141) | 125 (108 – 143) | < 0.001 |
| DBP (mmHg) | 64 (53 – 78) | 68 (57 – 80) | < 0.001 | 64 (53 – 78) | 68 (57 – 80) | < 0.001 |
| MAP (mmHg) | 82 (69 – 95) | 84 (72 – 97) | < 0.001 | 82 (69 – 95) | 84 (72 – 97) | < 0.001 |
| Heart rate (bpm) | 93 (79 – 111) | 89 (75 – 105) | < 0.001 | 93 (79 – 111) | 89 (75 – 105) | < 0.001 |
| Shock index | 0.78 (0.62 – 0.96) | 0.71 (0.57 – 0.89) | < 0.001 | 0.78 (0.62 – 0.96) | 0.71 (0.57 – 0.89) | < 0.001 |
| Respiratory rate (breaths/min) | 21 (16 – 27) | 19 (16 – 24) | < 0.001 | 21 (16 – 27) | 19 (16 – 24) | < 0.001 |
| S_p_O_2_ (%) | 98 (94 – 100) | 98 (95 – 99) | 0.3940 | 98 (94 – 100) | 97 (95 – 99) | 0.4211 |
| Temperature (C^o^) | 36 (36 – 37) | 36 (36 – 37) | < 0.001 | 36 (36 – 37) | 36 (36 – 37) | 0.0024 |
| GCS | 15 (13 – 15) | 15 (14 – 15) | < 0.001 | 14 (14 – 15) | 15 (14 – 15) | < 0.001 |
| Random glucose (mg/dL) | 137 (109 – 175) | 134 (107 – 180) | < 0.001 | 135 (107 – 172) | 134 (107 – 179) | < 0.001 |
| P_a_O_2_ (mmHg) | 103 (72 – 190) | 88 (74 – 102) | < 0.001 | 128 (78 – 167) | 107 (95 – 110) | < 0.001 |
| P_a_CO_2_ (mmHg) | 41 (34 – 50) | 40 (33 – 48) | < 0.001 | 44 (36 – 48) | 42 (38 – 44) | < 0.001 |
| HCO_3_^-^ (mmol/L) | 23 (19 – 26) | 23 (20 – 26) | < 0.001 | 23 (21 – 24) | 23 (21 – 25) | < 0.001 |
| Oxygen therapy (%) | 405 (17.7) | 2,467 (16.1) | 0.0616 | 405 (17.7) | 2,467 (16.1) | 0.0616 |
| Vasopressor (%) | 111 (4.8) | 419 (2.7) | < 0.001 | 111 (4.8) | 419 (2.7) | < 0.001 |

DBP, diastolic blood pressure; GCS, Glasgow Coma Scale; MAP, mean arterial pressure; SBP, systolic blood pressure.

**Supplementary Table S1B.** Baseline characteristics (mean) of cohort before and after missing data imputation

|  | Before Imputation | | | After Imputation | | |
| --- | --- | --- | --- | --- | --- | --- |
|  | Intubated  n = 2,292 | Non-intubated  n = 15,324 | *p* value | Intubated  n = 2,292 | Non-intubated  n = 15,324 | *p* value |
| Male (%) | 1,299 (56.7) | 8,301 (54.1) | 0.0261 | 1,299 (56.7) | 8,301 (54.1) | 0.0261 |
| Age (years) | 62 (52 – 74) | 60 (50 – 74) | 0.3576 | 63 (52 – 74) | 62 (50 – 74) | 0.3576 |
| SBP (mmHg) | 123 (104 – 141) | 127 (108 – 143) | < 0.001 | 123 (104 – 141) | 127 (108 – 143) | < 0.001 |
| DBP (mmHg) | 66 (53 – 78) | 69 (57 – 80) | < 0.001 | 66 (53 – 78) | 69 (57 – 80) | < 0.001 |
| MAP (mmHg) | 83 (69 – 95) | 86 (72 – 97) | < 0.001 | 83 (69 – 95) | 86 (72 – 97) | < 0.001 |
| Heart rate (bpm) | 96 (79 – 111) | 91 (75 – 105) | < 0.001 | 96 (79 – 111) | 91 (75 – 105) | < 0.001 |
| Shock index | 0.83 (0.62 – 0.96) | 0.75 (0.57 – 0.89) | < 0.001 | 0.83 (0.62 – 0.96) | 0.75 (0.57 – 0.89) | < 0.001 |
| Respiratory rate (breaths/min) | 22 (16 – 27) | 20 (16 – 24) | < 0.001 | 22 (16 – 27) | 20 (16 – 24) | < 0.001 |
| S_p_O_2_ (%) | 96 (94 – 100) | 97 (95 – 99) | 0.3940 | 96 (94 – 100) | 97 (95 – 99) | 0.4211 |
| Temperature (C^o^) | 36 (36 – 37) | 36 (36 – 37) | < 0.001 | 36 (36 – 37) | 36 (36 – 37) | 0.0024 |
| GCS | 13 (13 – 15) | 14 (14 – 15) | < 0.001 | 13 (14 – 15) | 14 (14 – 15) | < 0.001 |
| Random glucose (mg/dL) | 157 (109 – 175) | 165 (107 – 180) | < 0.001 | 154 (107 – 172) | 165 (107 – 179) | < 0.001 |
| P_a_O_2_ (mmHg) | 149 (72 – 190) | 109 (74 – 102) | < 0.001 | 150 (78 – 167) | 109 (95 – 110) | < 0.001 |
| P_a_CO_2_ (mmHg) | 45 (34 – 50) | 42 (33 – 48) | < 0.001 | 45 (36 – 48) | 42 (38 – 44) | < 0.001 |
| HCO_3_^-^ (mmol/L) | 23 (19 – 26) | 23 (20 – 26) | < 0.001 | 23 (21 – 24) | 23 (21 – 25) | < 0.001 |
| Oxygen therapy (%) | 405 (17.7) | 2,467 (16.1) | 0.0616 | 405 (17.7) | 2,467 (16.1) | 0.0616 |
| Vasopressor (%) | 111 (4.8) | 419 (2.7) | < 0.001 | 111 (4.8) | 419 (2.7) | < 0.001 |

DBP, diastolic blood pressure; GCS, Glasgow Coma Scale; MAP, mean arterial pressure; SBP, systolic blood pressure.

**Supplementary Table S2.** Feature importance of variables from random forest

| Feature Importance | Fold 1 | Fold 2 | Fold 3 | Fold 4 | Fold 5 | Fold 6 | Fold 7 | Fold 8 | Fold 9 | Fold 10 | Fold 11 | Fold 12 | Mean |
| --- | --- | --- | --- | --- | --- | --- | --- | --- | --- | --- | --- | --- | --- |
| Gender | 0.00 | 0.00 | 0.00 | 0.00 | 0.00 | 0.01 | 0.00 | 0.00 | 0.00 | 0.00 | 0.00 | 0.00 | 0.00 |
| Age | 0.02 | 0.02 | 0.02 | 0.01 | 0.02 | 0.02 | 0.02 | 0.03 | 0.02 | 0.02 | 0.02 | 0.02 | 0.02 |
| Heart rate | 0.02 | 0.02 | 0.01 | 0.02 | 0.03 | 0.02 | 0.02 | 0.03 | 0.01 | 0.02 | 0.03 | 0.02 | 0.02 |
| SBP | 0.01 | 0.01 | 0.01 | 0.01 | 0.01 | 0.01 | 0.01 | 0.02 | 0.01 | 0.01 | 0.01 | 0.01 | 0.01 |
| DBP | 0.01 | 0.02 | 0.01 | 0.01 | 0.01 | 0.01 | 0.01 | 0.02 | 0.01 | 0.01 | 0.01 | 0.01 | 0.01 |
| MAP | 0.01 | 0.01 | 0.01 | 0.01 | 0.01 | 0.01 | 0.01 | 0.01 | 0.01 | 0.01 | 0.01 | 0.01 | 0.01 |
| Respiratory rate | 0.04 | 0.03 | 0.04 | 0.04 | 0.04 | 0.04 | 0.05 | 0.06 | 0.03 | 0.04 | 0.04 | 0.04 | 0.04 |
| Temperature | 0.04 | 0.03 | 0.03 | 0.03 | 0.03 | 0.03 | 0.03 | 0.04 | 0.03 | 0.04 | 0.04 | 0.04 | 0.03 |
| S_p_O_2_ | 0.03 | 0.03 | 0.02 | 0.02 | 0.03 | 0.02 | 0.02 | 0.03 | 0.02 | 0.02 | 0.02 | 0.03 | 0.02 |
| GCS | 0.12 | 0.09 | 0.10 | 0.21 | 0.11 | 0.12 | 0.19 | 0.10 | 0.15 | 0.10 | 0.09 | 0.08 | 0.12 |
| Shock index | 0.02 | 0.03 | 0.02 | 0.03 | 0.03 | 0.03 | 0.03 | 0.04 | 0.02 | 0.02 | 0.02 | 0.03 | 0.03 |
| Pulse pressure | 0.01 | 0.01 | 0.01 | 0.01 | 0.01 | 0.01 | 0.01 | 0.01 | 0.01 | 0.01 | 0.01 | 0.01 | 0.01 |
| Glucose | 0.01 | 0.01 | 0.02 | 0.01 | 0.01 | 0.01 | 0.01 | 0.02 | 0.01 | 0.01 | 0.02 | 0.01 | 0.01 |
| P_a_O_2_ | 0.49 | 0.43 | 0.42 | 0.40 | 0.46 | 0.34 | 0.37 | 0.36 | 0.46 | 0.43 | 0.44 | 0.42 | 0.42 |
| P_a_CO_2_ | 0.10 | 0.12 | 0.18 | 0.12 | 0.12 | 0.21 | 0.12 | 0.15 | 0.15 | 0.16 | 0.10 | 0.13 | 0.14 |
| HCO_3_^-^ | 0.05 | 0.11 | 0.07 | 0.07 | 0.06 | 0.09 | 0.07 | 0.05 | 0.04 | 0.08 | 0.14 | 0.11 | 0.08 |
| Vasopressor | 0.00 | 0.00 | 0.00 | 0.00 | 0.00 | 0.00 | 0.00 | 0.00 | 0.00 | 0.00 | 0.00 | 0.00 | 0.00 |
| Oxygen therapy | 0.01 | 0.02 | 0.01 | 0.01 | 0.01 | 0.02 | 0.02 | 0.03 | 0.00 | 0.02 | 0.02 | 0.03 | 0.02 |
| Total | 1.00 | 1.00 | 1.00 | 1.00 | 1.00 | 1.00 | 1.00 | 1.00 | 1.00 | 1.00 | 1.00 | 1.00 | 1.00 |

Higher values represent higher importance of variable from random forest; DBP, diastolic blood pressure; GCS, Glasgow Coma Scale; MAP, mean arterial pressure; SBP, systolic blood pressure.

**Supplementary Table S3.** SHAP values of variables in random forest

| Mean(\|SHAP\|) | Fold 1 | Fold 2 | Fold 3 | Fold 4 | Fold 5 | Fold 6 | Fold 7 | Fold 8 | Fold 9 | Fold 10 | Fold 11 | Fold 12 | Mean |
| --- | --- | --- | --- | --- | --- | --- | --- | --- | --- | --- | --- | --- | --- |
| Gender | 0.00 | 0.01 | 0.01 | 0.00 | 0.00 | 0.01 | 0.00 | 0.01 | 0.01 | 0.00 | 0.00 | 0.01 | 0.01 |
| Age | 0.02 | 0.02 | 0.01 | 0.01 | 0.02 | 0.01 | 0.01 | 0.02 | 0.01 | 0.02 | 0.01 | 0.02 | 0.01 |
| Heart rate | 0.02 | 0.02 | 0.02 | 0.02 | 0.03 | 0.02 | 0.02 | 0.03 | 0.02 | 0.03 | 0.03 | 0.03 | 0.02 |
| SBP | 0.01 | 0.01 | 0.01 | 0.01 | 0.01 | 0.01 | 0.01 | 0.01 | 0.01 | 0.01 | 0.01 | 0.01 | 0.01 |
| DBP | 0.01 | 0.03 | 0.02 | 0.01 | 0.02 | 0.02 | 0.02 | 0.03 | 0.03 | 0.01 | 0.02 | 0.01 | 0.02 |
| MAP | 0.01 | 0.01 | 0.01 | 0.01 | 0.01 | 0.01 | 0.01 | 0.01 | 0.01 | 0.01 | 0.01 | 0.01 | 0.01 |
| Respiratory rate | 0.06 | 0.05 | 0.06 | 0.06 | 0.07 | 0.05 | 0.06 | 0.07 | 0.05 | 0.05 | 0.05 | 0.05 | 0.06 |
| Temperature | 0.04 | 0.03 | 0.03 | 0.03 | 0.04 | 0.03 | 0.03 | 0.04 | 0.04 | 0.04 | 0.04 | 0.04 | 0.04 |
| S_p_O_2_ | 0.03 | 0.03 | 0.03 | 0.02 | 0.03 | 0.03 | 0.02 | 0.03 | 0.03 | 0.03 | 0.02 | 0.03 | 0.03 |
| GCS | 0.17 | 0.12 | 0.15 | 0.25 | 0.15 | 0.17 | 0.19 | 0.14 | 0.18 | 0.14 | 0.12 | 0.10 | 0.16 |
| Shock index | 0.03 | 0.03 | 0.03 | 0.03 | 0.04 | 0.04 | 0.04 | 0.06 | 0.03 | 0.03 | 0.03 | 0.04 | 0.03 |
| Pulse pressure | 0.00 | 0.00 | 0.00 | 0.01 | 0.00 | 0.00 | 0.00 | 0.01 | 0.01 | 0.01 | 0.01 | 0.01 | 0.01 |
| Glucose | 0.01 | 0.01 | 0.01 | 0.01 | 0.01 | 0.01 | 0.01 | 0.01 | 0.01 | 0.01 | 0.01 | 0.01 | 0.01 |
| P_a_O_2_ | 0.39 | 0.36 | 0.31 | 0.30 | 0.31 | 0.27 | 0.30 | 0.29 | 0.36 | 0.30 | 0.36 | 0.35 | 0.32 |
| P_a_CO_2_ | 0.11 | 0.14 | 0.20 | 0.13 | 0.17 | 0.23 | 0.16 | 0.15 | 0.14 | 0.18 | 0.12 | 0.13 | 0.15 |
| HCO_3_^-^ | 0.08 | 0.11 | 0.10 | 0.10 | 0.09 | 0.09 | 0.09 | 0.07 | 0.06 | 0.11 | 0.15 | 0.12 | 0.10 |
| Vasopressor | 0.00 | 0.00 | 0.00 | 0.00 | 0.00 | 0.00 | 0.00 | 0.00 | 0.00 | 0.00 | 0.00 | 0.00 | 0.00 |
| Oxygen therapy | 0.01 | 0.01 | 0.01 | 0.01 | 0.01 | 0.02 | 0.01 | 0.03 | 0.01 | 0.02 | 0.02 | 0.03 | 0.02 |
| Total | 1.00 | 1.00 | 1.00 | 1.00 | 1.00 | 1.00 | 1.00 | 1.00 | 1.00 | 1.00 | 1.00 | 1.00 | 1.00 |

Higher values represent higher importance of variable in random forest; DBP, diastolic blood pressure; GCS, Glasgow Coma Scale; MAP, mean arterial pressure; SBP, systolic blood pressure.

**Supplementary Table S4.** Model performance with and without imputation

| Imputation | Classifier | AUC | Specificity | Sensitivity | NPV | PPV | NLR | PLR |
| --- | --- | --- | --- | --- | --- | --- | --- | --- |
| Cohort without any missing data* | LR | 0.68 | 0.62 | 0.66 | 0.65 | 0.64 | 0.55 | 1.76 |
|  | RF | 0.67 | 0.37 | 0.83 | 0.68 | 0.57 | 0.47 | 1.31 |
| KNN | LR | 0.72 | 0.56 | 0.76 | 0.70 | 0.63 | 0.44 | 1.71 |
|  | RF | 0.74 | 0.56 | 0.80 | 0.74 | 0.64 | 0.36 | 1.80 |
| AE | LR | 0.77 | 0.61 | 0.79 | 0.75 | 0.67 | 0.34 | 2.03 |
|  | RF | 0.86 | 0.66 | 0.88 | 0.85 | 0.73 | 0.18 | 2.72 |

AE, Autoencoder; AUC, area under the curve; KNN, K-nearest neighbors; LR, Logistic Regression; NLR, negative likelihood ratio; NPV, negative predictive value; PLR, positive likelihood ratio; PPV, positive predictive value; RF, Random Forest.

*Cohort of 2,345 patients with full set of dataset (gender, age, heart rate, systolic blood pressure, diastolic blood pressure, mean blood pressure, respiratory rate, temperature, S_p_O_2_, GCS, shock index, pulse pressure, glucose, P_a_O_2_, P_a_CO_2_, HCO_3_^-^, vasopressor, oxygen therapy).

**Supplementary Table S5.** Model performance comparison of random forest based on threshold

| Threshold | Accuracy | Specificity | Sensitivity | NPV | PPV | NLR | PLR |
| --- | --- | --- | --- | --- | --- | --- | --- |
| 0.60 | 0.73 | 0.91 | 0.54 | 0.67 | 0.85 | 0.50 | 6.27 |
| 0.50 | 0.77 | 0.81 | 0.73 | 0.75 | 0.80 | 0.33 | 4.09 |
| 0.40 | 0.77 | 0.66 | 0.88 | 0.85 | 0.73 | 0.18 | 2.72 |
| 0.30 | 0.71 | 0.46 | 0.96 | 0.91 | 0.64 | 0.09 | 1.83 |

NLR, negative likelihood ratio; NPV, negative predictive value; PLR, positive likelihood ratio; PPV, positive predictive value.

**Supplementary Table S6.** Model performance comparison of random forest with and without surgical patients

|  | AUC | Accuracy | Specificity | Sensitivity | NPV | PPV | NLR | PLR |
| --- | --- | --- | --- | --- | --- | --- | --- | --- |
| Cohort with all patients | 0.86 | 0.77 | 0.66 | 0.88 | 0.85 | 0.73 | 0.18 | 2.72 |
| Cohort with only non-surgical patients | 0.86 | 0.77 | 0.65 | 0.90 | 0.86 | 0.72 | 0.16 | 2.58 |

AUC, area under the curve; NLR, negative likelihood ratio; NPV, negative predictive value; PLR, positive likelihood ratio; PPV, positive predictive value.
